# A186 BALLOON-ASSISTED ENDOSCOPY IN THE MANAGEMENT OF SMALL BOWEL CROHN’S DISEASE – AN OVERALL EXPERIENCE FROM A CANADIAN TERTIARY CARE HOSPITAL

**DOI:** 10.1093/jcag/gwae059.186

**Published:** 2025-02-10

**Authors:** M Fazal, J C Bowron, M Reeson, S Zepeda-Gomez, B Halloran

**Affiliations:** Medicine, University of Alberta Faculty of Medicine & Dentistry, Edmonton, AB, Canada; Medicine, University of Alberta Faculty of Medicine & Dentistry, Edmonton, AB, Canada; University of Alberta, Edmonton, AB, Canada; Medicine, University of Alberta Faculty of Medicine & Dentistry, Edmonton, AB, Canada; Medicine, University of Alberta Faculty of Medicine & Dentistry, Edmonton, AB, Canada

## Abstract

**Background:**

Crohn’s disease (CD) is a chronic inflammatory disease of the gastrointestinal tract with a wide spectrum of gastrointestinal symptoms and disease complications including strictures, fistulae, malabsorption etc. Balloon-Assisted Endoscopy (BAE) at our institution plays a vital role in managing small bowel Crohn’s disease, to which conventional endoscopy has limited access. It allows better diagnostic and therapeutic abilities through better visualization, biopsies, and endoscopic balloon dilations of strictures.

**Aims:**

Our aim is to evaluate the efficacy and safety of Balloon-Assisted Endoscopy, including Single-Balloon Endoscopy (SBE) and Double-Balloon Endoscopy (DBE), in patients with Small Bowel Crohn’s disease by analyzing the demographics, indications, complications, and endoscopic therapies performed.

**Methods:**

Retrospective analysis of 231 patients who underwent a total of 532 BAE procedures between April 2012 – January 2024 at the University of Alberta Hospital (a tertiary care referral centre). The cohort included both DBE (344) and SBE (188) procedures and clinical findings and outcomes were analyzed.

**Results:**

A total of 532 BAE procedures (344 DBE, 188 SBE) were performed on 231 patients during April 2012 – January 2024. Out of 231 patients, 126 (54.5%) were males and the mean age of the cohort was 52.96 years. The mean duration of disease was 17.55 years and 109 patients (47.18%) had history of surgical resection before their first DBE. 94 patients (40.6%) underwent diagnostic DBE out of which 56 patients had single and 38 patients had multiple endoscopies. Whereas, 137 patients (59.3%) underwent therapeutic DBE with 60 patients undergoing single and 77 patients requiring multiple therapeutic procedures. Obstructive symptoms were reported before 96 procedures (18%). We were able to diagnose Adenocarcinoma (3 patients), Meckel’s Diverticulum (2 patients), retrieve Video-Capsule (1 patient) and achieve hemostasis via hemoclips in 4 patients. A total of 398 strictures were encountered out of which 16 (4.02%) were non-traversable and 3 (0.75%) were failed dilations. We had a > 95% successful dilation rate. Adverse events included aspiration leading to termination of procedure (n=3, 0.56%), mucosal/submucosal tears (n=4, 0.75%), bleeding requiring clips (n=3, 0.56%) and perforation requiring emergent surgery (n=1, 0.18%).

**Conclusions:**

We are reporting on one of the largest single center cohorts of BAE done in IBD patients in the literature. Overall, BAE has a strong safety profile in retrospective analysis of our cohort with a high success rate in both assessing disease and dilating the majority of strictures encountered. 40.6 % of our patients benefited from BAE as an ongoing diagnostic modality: assessing disease status and response to therapy.

**Funding Agencies:**

None

